# The prevalence of HBV, HCV and HIV infections among Iranian infertile couples referring to Royan institute infertility clinic: A cross-sectional study

**Published:** 2018-09

**Authors:** Mehrangiz Zangeneh, Maryam Sedaghat Jou, Mohammad Ali Sadighi Gilani, Mahin Jamshidi Makiani, Shaghayegh Sadeghinia, Reza Salman Yazdi

**Affiliations:** 1 *Department of Infectious Diseases, Tehran Medical Sciences Branch, Islamic Azad University, Tehran, Iran.*; 2 *Department of Andrology, Reproductive Biomedicine Research Center, Royan Institute for Reproductive Biomedicine, ACECR, Tehran, Iran.*; 3 *General Physician, Tehran Medical Sciences Branch, Islamic Azad University, Tehran, Iran.*; 4 *Department of Infectious Diseases, Iran University of Medical Sciences, Tehran, Iran.*; 5 *Department of Biomolecular & Biomedical Science, School of Health Life & Science, Glasgow Caledonian University, Glasgow, UK.*

**Keywords:** HBsAg, HCV antibody, HIV antibody, Infertility, Viral hepatitis

## Abstract

**Background:**

The role of the screening protocol for viral hepatitis and human immuunodeficiency virus (HIV) infections among infertile couples were seldom investigated.

**Objective:**

The present study was performed to assess the prevalence of hepatitis B virus (HBV), hepatitis C virus (HCV) and HIV infections among infertile couples referring to infertility clinic of Royan Institute.

**Materials and Methods:**

This analytical cross-sectional study was performed on 21673 infertile couples referring to infertility clinic of Royan Institute between 2009 and 2014. Serological findings for viral hepatitis B, C and HIV infection were gathered herewith demographic data of the study participants through the study checklist. Ultimately, 302 couples who had at least one positive result in their serological tests were included in the statistical analysis.

**Results:**

The HBV and HCV infections prevalence among study participants were 0.57% and 0.148% respectively; only two cases had HIV infection. HBV and HCV infections prevalence had significant association with the gender of participants, but there was no significant relationship between these infections and infertility types.

**Conclusion:**

Viral hepatitis infections screening among infertile couples undergoing assisted reproductive techniques needs more attention.

## Introduction

An infertile couple was defined as a couple which had not had positive pregnancy experience after one-year regular unprotected sexual intercourse without contraceptive drugs use or other methods. According to world health organization reports, there are more than 80 million infertile couples around the world specially in the developing countries, and infertility prevalence among Iranian couples is 17.2% (1). Among Iranian population, the prevalence of hepatitis B (HBV) and hepatitis C (HCV) virus infection were estimated at about 2.14% and 0.16%, respectively (2, 3). Severe pathological complications such as viral cirrhosis or hepatocellular carcinoma and even death are considered as the sequelae of chronic hepatitis infection (4).

The impact of viral infection on the outcome of Assisted Reproductive Techniques (ART) among infertile couples is one of the interesting controversial research topics in reproductive medicine. Some investigators believe that semen quality among infertile couples is lower than normal reference limits (5, 6), while some others report no difference between them (7-9). It seems that immunological conditions and antiviral therapy in patients with hepatitis or human immunodeficiency virus (HIV) infection can lead to hypogonadism and decrease sperm quality (10, 11).

In one study on 712 infertile couples in Ahvaz city (in south west of Iran), investigators reported that HBV and HCV were positive in 11 (0.77%) and 9 (0.63%) couples, respectively (12). Passos and colleagues assessed 409 infertile couples in a Brazilian infertility clinic and reported that the HCV infection rate among male and female infertile patients were 3.2% and 3.7% respectively. They concluded that HCV screening test had been necessary for all of infertile couples who were ART candidates (13).

Different fertility related factors including marital age, multiple sex partners, environmental pollution, alcohol consumption, smoking and infectious disorders among societies cause different infertility assessment results among countries (14). It seems that the accurate screening data of Iranian infertile couples is necessary for assessing the role of HBV and HCV infection on infertility rate and ART outcomes among them.

In this context, the present study was performed for evaluation of HBV, HCV and HIV infection prevalence among Iranian infertile couples who were ART candidate in the Royan infertility clinic between 2009 and 2014. 

## Materials and methods

**Study design**

This analytical cross-sectional study had been performed for assessment of HCV, HBV and HIV infection prevalence among Iranian infertile couples who referred to Royan Institute infertility clinic for treatment of infertility between March 2009 and April 2014. Inclusion criteria for this study were every couple who had the results of the serological tests for hepatitis B surface antigen (HBsAg), HCV antibody and HIV antibody in their medical folders. There were no exclusion criteria for these couples in this study.

**Study population and data collection**

The study population consists of the infertile couples who had positive laboratory findings for one of the viral hepatitis B or hepatitis C or HIV. Demographic variables such as age, sex, and infertility types were gathered with laboratory findings of infertile couples including, HBsAg, HCV antibody and HIV antibody, in the data checklist. For statistical analysis, the study participants were divided into six age groups including less than 20, 21-25, 26-30, 31-35, 36-40 and older than 41 yr old. All of the serological tests were performed in the clinical laboratory of Royan Institute using ELISA method and approved commercial kits (Pishtaz-Teb Inc. Tehran, Iran).

**Ethical consideration**

Research and Medical Ethics Committee of the Royan Institute approved the present study protocol (REC.1396.263) and all the study participants signed the consent forms for study.

**Statistical analysis**

All statistical analyses were performed using the Statistical Package for the Social Sciences, Version 21.0 software (SPSS, Inc., Chicago, Illinois, USA). Quantitative and qualitative variables were presented as mean (standard deviation) and frequency (percentage), respectively. Normality of distribution frequency of study variables was assessed by ANOVA (Analysis of variances). In the univariate statistical analysis for normal variables, independent sample t-test was used. Chi-square test was used for determination of significant association between study qualitative variables. All of p≤0.05 were assumed as significant results.

## Results

In the study period, 43274 persons (21637 couples) were referred to the Institute and among them, 604 individuals (302 couples, 1.39%) had at least one positive finding for HBV, HCV or HIV infection. The following statistical analysis was performed on the 302 couples with positive findings. Most of the study participants’ couples were in 31-35 (31.6%) and 36-40 (21.9%) age groups, respectively. Most infertile females were in the 31-35 and more than 41-yr old age group (each 31.1%), while the most infertile males were in the 31-35 yr old group (32.1%) (Table I). The frequency of primary infertility among study participants was higher than secondary infertility types (447, 74% vs. 157, 26%).

HBV infection prevalence among the study population was 0.57% (247 individuals). The prevalence of HBV infection in males was significantly higher than female participants (176, 0.81% vs. 71, 0.33%; respectively, p≤0.001). HCV infection prevalence among infertile couples was 0.148% (64 individuals). Male participants had significantly higher HCV infection prevalence than females (58, 0.27% vs. 6, 0.01%; respectively, p<0.001). Only two infertile male participants (about 0.0046%) had positive HIV antibody test in the serological assessment (Table II).

The prevalence of HBV infection in 31-35 and >41 yr old age groups were 32.79 % and 25.1%, respectively; more than the other age groups. HBV infection prevalence had a significant association with the age group of study participants (p≤0.001). In this manner, the prevalence of HCV infection was higher among the participants of >41 (35.94%) and 31-35 (29.69%) yr old age groups too, and there was a significant association between HCV infection prevalence and age groups (p≤0.001) (Table III).

Prevalence of HBV infection among participants with primary and secondary infertility types were 0.4% and 0.1%, respectively. Although frequency of HBV infection was higher among participants with primary infertility, this difference was not statistically significant (p=0.42). Prevalence of HCV infection among participants with primary and secondary infertility types were 0.1% and 0.04%, respectively. Prevalence of HCV infection did not have a significant association with infertility types (p=0.43).

**Table I:** Prevalence of age and sex distribution among study participants

**Age groups (yr)**	**Sex**	
	**Male**	**Female**
<20	2 (0.7%)	0 (0%)
21-25	42 (13.9%)	6 (2%)
26-30	71 (23.5%)	38 (12.6)
31-35	97 (32.1%)	94 (31.1%)
36-40	62 (20.5%)	70 (23.2%)
>41	28 (9.3%)	94 (31.1%)
Total	302 (100%)	302 (100%)

Data presented as n (%)

**Table II:** Prevalence of HBV, HCV and HIV infection among infertile groups according to their gender

		**Female**	**Male**
HBsAg			
	Positive	71 (23.51%)	176 (58.67%)
	Negative	231 (76.49%)	124 (41.33%)
	Total	302 (100%)	300 (100%)
HCV infection			
	Positive	6 (2.0%)	58 (19.46%)
	Negative	295 (98%)	240 (80.54%)
	Total	301 (100%)	298 (100%)
HIV infection			
	Positive	0 (0%)	2 (0.7%)
	Negative	301 (100%)	296 (99.3%)
	Total	301 (100%)	298 (100%)

Data presented as n (%)

HBsAg: Hepatitis B surface antigen

HCV: Hepatitis C virus

HIV: Human immunodeficiency virus

**Table III:** Frequency of HBV and HCV infection among infertile groups according their age groups

**Age groups (yr)**	**HBsAg**		**HCV infection**	
	**Positive**	**Negative**	**Positive**	**Negative**
<20	0 (0%)	2 (0.57%)	0 (0%)	2 (0.37%)
21-25	9 (3.65%)	39 (10.99%)	1 (1.74%)	47 (8.78%)
26-30	45 (18.22%)	64 (18.03)	7 (10.94%)	101 (18.88%)
31-35	81 (32.79%)	109 (30.70%)	19 (29.69%)	170 (31.78%)
36-40	50 (20.24%)	82 (23.09%)	14 (21.69%)	117 (21.87%)
>41	62 (25.10%)	59 (16.62%)	23 (35.94%)	98 (18.32%)
Total	247 (100%)	355 (100%)	64 (100%)	535 (100%)

Data presented as n (%)

HBsAg: Hepatitis B surface antigen

HCV: Hepatitis C virus

## Discussion

The findings of the present study showed that HBV, HCV and HIV infections prevalence among study population were 0.57%, 0.148% and 0.0046%, respectively. HBV and HCV infections had a significant association with age of infertile couples, and they were significantly more prevalent in males than females, as well.

Searching in the literature showed us that there were some similar studies with the same objectives as our study. Nikbakht and colleagues performed their study among 712 infertile couples in Khuzestan province in the west southern of Iran and reported 11 and 9 infertile couples who had HBV and HCV infection, respectively (12). They believed that low prevalence of hepatitis infection in their study is related to their low sample size and suggested next studies with higher sample size in national scale in which including more Iranian provinces (12, 15-16). 

Salman Roughani and colleagues performed their study on 1230 blood donors in Yazd city in the south of Iran to determine HBV and HCV infection prevalence and reported that 15.1% of blood donors had positive HBc antibody and among them, 51.6% had positive HBs antibody. They suggested next studies with more details for prevention of infected blood samples with hepatitis virus transfusion (17). In a recent systematic review by Alavian and colleagues on the prevalence of HBV infection among Iranian population, the prevalence of HBsAg-positive individuals was higher in Golestan, Tehran and Hormozgan provinces, respectively (2). 

Cheraghali and colleagues performed their study on 1553 pregnant women who were referred to Deziani hospital for following their treatment protocol and reported the prevalence of HBV infection among pregnant women was 1%, and suggested that the pregnant women must be screened for HBV infection in the first trimester (18). Sharifi and colleagues in their study on 323 pregnant women reported 11 women with positive HBsAg that none of them were HBeAg positive (19). Bani Aghil and colleagues in their study reported that the prevalence of HBV, HCV and HIV infection among blood donors in Golestan province in the north of Iran were 1.25%, 0.14% and 0.0015%, respectively. They also noted the higher prevalence in male blood donors with lower educational level (20).

In our study on 21637 couples, 247 and 64 infertile couples were positive for HBV and HCV infection respectively. Searching through the literature, we did not find similar studies with high sample size; therefore, we can rely on the study findings. Although similar studies were performed with the same purposes among Iranian population specially blood donors, most of them had the low sample size and only reported hepatitis prevalence, and they have not reported prevalence of viral hepatitis in different age and sex categories and infertile couples. 

In this study, we have investigated more than 20,000 infertile couples who were referred to one of the main infertility clinics in Iran and even Middle East countries. In addition to total prevalence of viral hepatitis and HIV in infertile couples, we reported HBV and HCV infection frequencies among different age and sex categories and we assessed the relationship of age and sex with the hepatitis infections among study participants.

In conclusion, we suggest that the viral hepatitis infections screening among infertile couples who are undergoing assisted reproductive techniques is an important subject and needs more attention.

**Limitation**

Our study had some limitations: First, the study participants were from one infertility clinic, and it may have impact on the study results; next studies with randomly infertile couples from all of the Iranian infertility clinics is suggested. Second, most of our data in the present study were on the base of medical folders of infertile couples and some of medical folders did not have enough data. Third, it is suggested next studies to compare hepatitis infection among infertile and non-infertile couples.

## Note

***This article extracted from M.D. Thesis. (Maryam Sedaghat Jou)***
